# Supplementary material for: A Century of Swine Influenza: Is It Really Just about the Pigs?
Source: Vet Sci. 2020 Nov 26;7(4):189. doi: 10.3390/vetsci7040189 (PMC7711507; doi:10.3390/vetsci7040189)
Supplement: Supplementary file 1 [file vetsci-07-00189-s001.pdf]

Supplementary Materials

Table S1. Detailed search criteria applied to search on the Scopus database.

Group	Search Criteria
Swine	TITLE-ABS-KEY (Swine AND flu OR swine AND influenza) AND (porcine AND flu OR porcine AND influenza) AND (pig AND flu OR pig AND influenza)
Human	TITLE-ABS-KEY (Swine AND flu OR swine AND influenza) AND (porcine AND flu OR porcine AND influenza) AND (pig AND flu OR pig AND influenza) AND (human) AND (variant)
Mice	TITLE-ABS-KEY (Swine AND flu OR swine AND influenza) AND (porcine AND flu OR porcine AND influenza) AND (pig AND flu OR pig AND influenza) AND (mice variant OR mouse variant)
Ferrets	TITLE-ABS-KEY (ferret OR ferrets) AND (swine AND flu OR swine AND influenza) AND (porcine AND flu OR porcine AND influenza) AND (pig AND flu OR pig AND influenza)
H_4N_6	#1 minus #2, where:<br>#1 = TITLE-ABS-KEY (Swine OR pig OR porcine) AND (H1N1 OR h1n2 OR h3n1 OR h3n2 OR h2n3 OR h1n7 OR H4N6) AND (virus) AND (variant)<br>#2 = TITLE-ABS-KEY (Swine OR pig OR porcine) AND (H1N1 OR h1n2 OR h3n1 OR h3n2 OR h2n3 OR h1n7) AND (virus) AND (variant)
H_1N_7	#2 minus #3, where:<br>#3 = TITLE-ABS-KEY (Swine OR pig OR porcine) AND (H1N1 OR h1n2 OR h3n1 OR h3n2 OR h2n3) AND (virus) AND (variant)
H_2N_3	#3 minus #4, where:<br>#4 = TITLE-ABS-KEY (Swine OR pig OR porcine) AND (H1N1 OR h1n2 OR h3n1 OR h3n2) AND (virus) AND (variant)
H_3N_2	#4 minus #5, where:<br>#5 = TITLE-ABS-KEY (Swine OR pig OR porcine) AND (H1N1 OR h1n2 OR h3n1) AND (virus) AND (variant)
H_3N_1	#5 minus #6, where:<br>#6 = TITLE-ABS-KEY (Swine OR pig OR porcine) AND (H1N1 OR h1n2) AND (virus) AND (variant)
H_1N_2	#6 minus #7, where:<br>#7 = TITLE-ABS-KEY (Swine OR pig OR porcine) AND (H1N1) AND (virus) AND (variant)
H_1N_1	#7<br>TITLE-ABS-KEY (Swine OR pig OR porcine) AND (H1N1) AND (virus) AND (variant)

Table S2. Top 20 countries with highest scientific production measured as number of publications produced during the studied time-period (1930-2020). The rest of the countries (non-listed) range from 1 to 35 publications.

Country	Articles
USA	1085
CHINA	310
GERMANY	303
FRANCE	226
JAPAN	174
UK	173
SPAIN	151
CANADA	139
BELGIUM	123
ITALY	117
NETHERLANDS	104
DENMARK	71
BRAZIL	64
THAILAND	60
SOUTH KOREA	57
POLAND	56
MEXICO	46
HUNGARY	41
SWITZERLAND	37
AUSTRIA	35

Table: S3. Top 20 countries with highest number of international collaborations counted as number of countries in their collaboration network during the studied time period (1930-2020).

Country	Number of Countries (Collaborations)
USA	72
UNITED KINGDOM	56
CHINA	41
CANADA	39
SPAIN	39
FRANCE	36
NETHERLANDS	34
JAPAN	32
AUSTRALIA	29
BELGIUM	28
SWEDEN	28
ITALY	24
DENMARK	17
BRAZIL	16
CZECH REPUBLIC	16
SWITZERLAND	16
GERMANY	15
INDIA	15
THAILAND	15
ARGENTINA	12

Table: S4. DOIs analyzed via manual screening along with the GenBank IDs mentioned on each of them.

DOI	Genbank ID
http://doi.org/10.1038/EMI.2012.33	JX534958-JX534973
http://doi.org/10.1038/EMI.2012.38	ACF22215,CY103965 (NS-124I),CY103967 (NS-125G),CY103966 (NS-124I+125)
http://doi.org/10.1099/VIR.0.045765-0	JX568153-JX568158,AB126266
http://doi.org/10.1016/J.VIROL.2012.08.003	CY046940,CY046941,CY046942,CY039527CY046943,CY039528CY046944,CY046945,CY077600,CY077601,CY077602,CY077603,CY077604,CY077605,CY077606,CY077607,CY077608,CY077609,CY077610
http://doi.org/10.1016/J.VIRUSRES.2012.07.025	AB704796-AB704859
http://doi.org/10.1371/JOURNAL.PONE.0045946	FJ966082,FJ966084,CY045226,CY045234CY045242,CY047744CY053474,CY053482,CY053490,CY053498,CY053506,JN381203-JN381340,CY045228,CY045236,CY045244,CY047746,CY053476,CY053484,CY053492
http://doi.org/10.1371/JOURNAL.PONE.0041866	EPI355210-EPI355228
http://doi.org/10.1016/J.VETMIC.2012.01.021	EF635006
http://doi.org/10.1016/J.VIRUSRES.2012.04.014	FJ157986
http://doi.org/10.2183/PJAB.88.226	BAA01280,CBA17655,ABV25634,AAM75158ABD62843,ABP49316ABF21277,ABO38395,AAP34322,AAK70453,ACA28844,AAD17229,ACP41105
http://doi.org/10.1128/JVI.07242-12	JN582051-JN582066
http://doi.org/10.1128/JVI.07158-11	AAA64362,JN130388-JN130397,CY044325
http://doi.org/10.1016/J.VIRUSRES.2012.03.003	JQ858372-JQ858398
http://doi.org/10.1007/S11033-012-1530-2	AJ628065
http://doi.org/10.1111/J.1750-2659.2012.00337.X	CY080402,CY080437-449,CY080404.,HQ712177-183,HQ712184
http://doi.org/10.1371/JOURNAL.PONE.0036318	Table 2 within article
http://doi.org/10.1007/S11262-011-0704-7	JN809093–JN809236
http://doi.org/10.3892/MMR.2011.709	HM014332.1
http://doi.org/10.1128/JVI.06824-11	JQ220532-JQ220556
http://doi.org/10.1016/J.MCP.2011.10.003	FJ981615,GQ117032,GQ117119
http://doi.org/10.1371/JOURNAL.PONE.0029347	FJ951848–FJ951855
http://doi.org/10.1186/1297-9716-43-24	JF960169,JF960172 - JF960174,JF960176,JF960177JF960180-JF960184,JF960187JF960189,JF960190,JF960192,JF960193,JF960197,JF960199 - JF960208,JQ301920 - JQ301944

http://doi.org/10.1111/J.1750-2659.2011.00267.X	AB598480–AB598527
http://doi.org/10.1016/J.JVIROMET.2011.10.017	NC001792,HM143844<br>Table within article
http://doi.org/10.1186/1743-422X-8-537	AB620160- AB620211
http://doi.org/10.1371/JOURNAL.PONE.0025848	GQ283488,GU292341,HQ228034,HQ228039-40HQ228037,HQ228042HQ228053-4,HQ228057,HQ228059-60,HQ228067-8,HQ228074,HQ228076,HQ228078-80,HQ228083-4,HQ228086,HQ228090-1,HQ228093-6,HQ228098,HQ228103-4
http://doi.org/10.1128/JVI.05352-11	JN374994-JN375281
http://doi.org/10.1128/JVI.05262-11	JN375282-JN375321
http://doi.org/10.1371/JOURNAL.PNTD.0001342	JN378747,JN7378748
http://doi.org/10.1371/JOURNAL.PONE.0020130	AAT08000
http://doi.org/10.1128/JVI.02125-10	EU798918,EU798898,EU798878;,FJ009502FJ009455,FJ009455EU301177,EU301400,,EU301368,EU301207,EU301430,EU301398,EU233746,EU233745,EU233744,GU361390,GU361308,GU361350,HQ913050
http://doi.org/10.1074/JBC.M111.224469	X65018),U43092
http://doi.org/10.1007/S11262-011-0571-2	HM626479-HM626486,AF038270
http://doi.org/10.1371/JOURNAL.PONE.0018314	ISDN13422,CY001952,CY003833,CY002392CY006363,CY007467CY002624,CY003384,CY003304,CY003696,AF386780,AF386775,AY289928,AF386774,D13574,X17221,U02085,M38312,S62154
http://doi.org/10.1186/1743-422X-8-129	Table within article
http://doi.org/10.1074/JBC.M110.193557	FJ966082,AF455675,CY028163,40980.1ABB20429.1
http://doi.org/10.3201/EID1703.100851	FN423708–15,FR716024
http://doi.org/10.1007/S00239-010-9405-4	AAT08000
http://doi.org/10.1128/JVI.01694-10	CY054706,CY054705,CY054704,CY054699CY054702,CY054701CY054700,CY054703
http://doi.org/10.1093/GLYCOB/CWQ135	AP_003767.1,PM0188,NP_245125.1,AB293985.1, BC069151.1
http://doi.org/10.1371/JOURNAL.PONE.0013381	CY073781-CY073788,CY073789-CY073796,FJ966960 – FJ966965,FJ971074-FJ971075
http://doi.org/10.1371/JOURNAL.PONE.0012591	GU215015-GU215038
http://doi.org/10.1016/J.VIROL.2010.07.007	Table within article
http://doi.org/10.1016/J.VIROL.2010.06.018	AF117241
http://doi.org/10.1186/1743-422X-7-209	HM745138-HM745250

http://doi.org/10.1016/J.VACCINE.2010.07.065	AY626145.1,AY626144.1
http://doi.org/10.1016/J.CCA.2010.04.002	FJ966083
http://doi.org/10.1016/J.VIRUSRES.2010.03.019	FJ966976,FJ966978,FJ966977,FJ966974GQ338390,GQ377078FJ966975,FJ969538
http://doi.org/10.1080/10495391003617663	AB516285,L119P09
http://doi.org/10.1111/J.1863-2378.2009.01270.X	ABD77116
http://doi.org/10.1099/VIR.0.016691-0	AB514929–AB514943
http://doi.org/10.1016/J.VIROL.2009.12.014	GQ867592,GQ867595,GQ867593,GQ867594
http://doi.org/10.1371/JOURNAL.PONE.0009068	FJ969540,FJ966974,FJ969540,FJ981613
http://doi.org/10.1128/JVI.02106-09	FJ969540,CY022069,CY009628,EU516090CY030230,AF117241
http://doi.org/10.1128/JVI.00296-10	Table within article
http://doi.org/10.1186/1743-422X-6-180	GQ495129-GQ495136
http://doi.org/10.1016/J.DIAGMICROBIO.2009.07.016	Table within article
http://doi.org/10.1016/J.VACCINE.2009.07.040	ACP41107,ACP41105,ACA28847,ACA28844ACO95273,ACO95270ACA33351,ACA33493
http://doi.org/10.1016/J.BBRC.2009.05.056	Table within article
http://doi.org/10.1016/J.VETMIC.2009.03.010	FJ384744–FJ384759
http://doi.org/10.1007/S11434-009-0421-Y	Table within article
http://doi.org/10.1038/NATURE08182	GQ229259–GQ229378,GQ229259–GQ229378
http://doi.org/10.1128/JVI.02565-08	CY037895-CY038027
http://doi.org/10.1186/1743-422X-6-34	FJ688266,FJ688267,FJ688268,FJ688269
http://doi.org/10.1128/JVI.02403-08	FJ461592-FJ461607
http://doi.org/10.1016/J.VIRUSRES.2008.11.008	EU004440-EU004455<br>Table within article
http://doi.org/10.1128/JCM.01228-08	EU826543- EU826550,EU826551-EU826558
http://doi.org/10.1007/S11262-008-0251-Z	EU186411,EU224366,EU224368
http://doi.org/10.1016/J.VETMIC.2008.02.024	CY014613,CY005514,CY005511,CY005512CY005516,CY005515CY005513,CY006022

http://doi.org/10.1128/JCM.00398-08	CY030216,CY030217,CY030218,CY030215CY030235,CY030213CY030223,CY030219,CY030220,CY030222,CY030221,CY030224,CY030225,CY030226,CY030214,CY030227,CY030228,
http://doi.org/10.1128/JCM.01257-07	Table within article
http://doi.org/10.1128/JVI.01702-07	AF302264
http://doi.org/10.1007/S11262-007-0097-9	EF101754,EF101753,EF101755,EF101749EF101752,EF101756EF101750,EF101751
http://doi.org/10.1016/J.ANTIVIRAL.2007.03.007	EF409245–EF409258.,AJ311466,AJ291403,AJ252132AJ252130,AAT6472AAT6473,AAT6474
http://doi.org/10.1139/W07-044	AY747618
http://doi.org/10.1016/J.BBRC.2007.02.096	Table within article
http://doi.org/10.1016/J.VIRUSRES.2006.11.008	U47304,U53166,U53169,M63755U49485,DQ280258M25934,DQ139325
http://doi.org/10.1128/JVI.02458-06	DQ975252-DQ975267
http://doi.org/10.3201/EID1312.061323	DQ889682-DQ889689
http://doi.org/10.1128/JCM.44.3.1123-1126.2006	Q280221-DQ280228,DQ280229-DQ280236,DQ280205-DQ280212,DQ280245-DQ280252, DQ280237-DQ280244,DQ280189-DQ280196, DQ280197-DQ280204,DQ280260
http://doi.org/10.3201/EID1207.060268	DQ469955–DQ469962,DQ469963–DQ469970,DQ469971–DQ469978,DQ469979–DQ469986DQ469987–DQ469994,DQ469995–DQ470002DQ280256,DQ280253-DQ280255
http://doi.org/10.1128/JCM.42.9.4349-4354.2004	AY619970-AY619977,AY619962-AY619969,AY619954-AY619961,AY619979
http://doi.org/10.1128/JVI.78.17.9073-9083.2004	AY497328,AY497329,AY497330,AY497331M18379
http://doi.org/10.1128/JVI.78.1.240-249.2004	AF222810,AY428485-AY428504 ,AF222810
http://doi.org/10.1128/JVI.76.21.10717-10723.2002	AY130766,M54941
http://doi.org/10.1016/S0168-1702(02)00053-9	AY06003-52,AY060077-101,AY060053-76,AY060102-125AY060126-149,AY060150-173AY060174-197,AY060198-221
http://doi.org/10.1165/AJRCMB.26.6.4520	AF132496,X65018
http://doi.org/10.1098/RSTB.2001.0998	Figure within article
http://doi.org/10.1128/JVI.75.20.9679-9686.2001	AF222810,AF222825,AF400752,AF400791
http://doi.org/10.1128/JVI.75.20.9741-9752.2001	M22324,Z29522,U58920
http://doi.org/10.1016/S0264-410X(01)00190-6	Table within article
http://doi.org/10.1073/PNAS.031575198	M12597,AF333238

http://doi.org/10.1099/0022-1317-82-6-1397	AJ293920-AJ293943,AJ311454-AJ311466
http://doi.org/10.1016/S0168-1702(00)00154-4	AF251 389–AF251 394,AF251 395–AF251 402,AF251 403 – AF251 410,AF251 411–AF251 418AF251 419– AF251 426,AF25 1427–AF251 434
http://doi.org/10.1007/S007050070098	Table within article
http://doi.org/10.1128/JVI.74.19.9322-9327.2000	AF285885,AF285887,AF285886,AF285888AF285889,AF285890AF285891,AF285892,AF285883,AF285884
http://doi.org/10.1023/A:1008131312306	M63522,M63515-M63540,AF188003,AF188004
http://doi.org/10.1016/S0264-410X(98)00392-2	Table within article
http://doi.org/10.1007/S007050050415	Figure within article
http://doi.org/10.1016/S0168-1702(98)00038-0	M80942 – M80947,M80949 –M80978
http://doi.org/10.1016/S0168-1702(98)00011-2	Table within article
http://doi.org/10.1016/S0168-1702(98)00067-7	U77830
http://doi.org/10.1007/S007050050086	U3719,U3720,U80949,U80950, U80948,U11857,U11703,U11858,Z46436,U04859,Z46434,Z46441
http://doi.org/10.1016/S0923-2516(97)83632-1	U49722
http://doi.org/10.1099/0022-1317-76-10-2539	U11703,Ul1856,Ul1857,Ul1855U11858
http://doi.org/10.1016/0042-6822(91)90118-U	M55649,M55471,M55472,M55473.M55482,M55484,M55483
http://doi.org/10.1016/J.GENE.2019.03.044	NR_038549.1,NR_038518.1,NM_001206359.1,XM_005671132.1.,XM_003358827.3,M25302.1,NC_010445.3
http://doi.org/10.1111/IRV.12631	KY818816-KY818823
http://doi.org/10.1016/J.CELREP.2019.03.020	AF086833,ABX75367.1,AAU43887.1,ABA87127.1.ACI28629.1,KJ660346.2,AF086833.2,NC006432.1,KM655246.1,MK552329 – MK552374
http://doi.org/10.1016/J.MCP.2019.02.002	AF353511,KT591944,LC022792,KT199103, KY111278,MG334554
http://doi.org/10.3390/V11040377	KF425659–KF425665
http://doi.org/10.1073/PNAS.1809667116	MG999832-35,AAT84354.1,P15777.1,AAT84354.1,ASB17086.1,ADN03339.1,P11224.2
http://doi.org/10.1111/ZPH.12542	KM507541
http://doi.org/10.3390/V11020126	JQ315414.1, JQ315415.1, JQ315416.1, DQ095779.1
http://doi.org/10.1111/TBED.12998	Figure within article
http://doi.org/10.1128/JVI.00997-18	Figure within article
http://doi.org/10.1007/S12250-019-00140-1	Table within article
http://doi.org/10.1128/JVI.01982-18	KU820898
http://doi.org/10.1038/S41426-018-0088-Z	KM061010-KM061025, MF927789-MF927884

http://doi.org/10.1371/JOURNAL.PPAT.1007417	JX280447-JX280454, MH879773-MH879780
http://doi.org/10.1038/S41426-018-0109-Y	Figure within article
http://doi.org/10.1038/S41598-018-24371-6	Figure within article
http://doi.org/10.1007/S00705-018-4001-9	KT021232.1, AFO42860.1
http://doi.org/10.1016/J.JVIROMET.2018.09.009	LT727518, JF797217
http://doi.org/10.1038/S41426-018-0154-6	MH209304- 209311, MF630034-630041
http://doi.org/10.1371/JOURNAL.PONE.0208789	NZ_CP029003.1, NC_009053.1, ADOI00000000.1, ADOJ00000000, ADOK00000000.1, AF363361, KY468982, KY468983 ,KY468984, KY468985, KY468986, KY468987, KY468988, KY468989, KY468990, KY468991, KY468992 KY468993, KY468994
http://doi.org/10.1007/S13205-018-1427-4	MF564203–MF564216, NC_026433.1
http://doi.org/10.1128/JVI.01206-18	FJ969514.1
http://doi.org/10.1128/JVI.00316-18	MG962421– MG962468, MG962477–MG962508
http://doi.org/10.1186/S13567-018-0572-4	Table within article
http://doi.org/10.3389/FCIMB.2018.00240	HQ688673.1, HQ688675.1, MG812679, MG812678, HQ688673.1, DQ328874.1, DQ328875.1, AN20410 ,AY772170.1, AY772169.1
http://doi.org/10.1186/S12985-018-1013-8	GU569948, KR611515
http://doi.org/10.1371/JOURNAL.PONE.0199227	MF984401
http://doi.org/10.1016/J.PREVETMED.2018.02.019	Table within article
http://doi.org/10.1111/EVA.12536	Figures within article
http://doi.org/10.1111/ZPH.12378	KU321893-KU323320
http://doi.org/10.1016/J.VACCINE.2017.10.052	AF353511.1
http://doi.org/10.1038/S41598-017-01512-X	KY785904-KY785911
http://doi.org/10.1007/S00705-017-3548-1	KP728470.1
http://doi.org/10.1016/J.JVIROMET.2017.09.021	MF611876.1
http://doi.org/10.1093/INFDIS/JIX385	KC597139, KJ196286, DQ438972, KP698930
http://doi.org/10.1016/J.ANTIVIRAL.2017.09.009	MF001012-MF001019

http://doi.org/10.1016/J.VIROL.2017.08.024	EU258939, EU258937, JN401974, CY062485, CY103897, CY098760, KF021597.1, KF021599.1 ,KP307984.1,, KP307986.1
http://doi.org/10.1111/TBED.12547	EPI702901-EPI702908, EPI702959-EPI702966, EPI702264-EPI702271, EPI702169-EPI702176, EPI702676-EPI702683, EPI702185-EPI702192, EPI702177-EPI702184, EPI702609-EPI702616 ,EPI702347-EPI702354
http://doi.org/10.1016/J.RVSC.2017.05.008	KP229773
http://doi.org/10.1128/MSPHERE.00307-17	SRP102422
http://doi.org/10.1016/J.VETMIC.2017.06.009	KJ907484-KJ907491
http://doi.org/10.3201/EID2307.170019	Figures within article
http://doi.org/10.1007/S00705-017-3282-8	JN086908, CY077595, K00992, AF153234, EF566037.1, EF566075.1, AF222033.2, AF251427.2 ,AY289929.1, DQ487340, CY115996
http://doi.org/10.1371/JOURNAL.PONE.0175267	KY785881-KY85903, AF156435.1, AF156421.1, AF156449.1, AF156378.1, AF156407.1, AF156396.1, AF156463.1 ,AF156477.2, KY785896-KY785903
http://doi.org/10.3390/IJMS18040749	JF275925-JF275932, EU594346-EU594353, DQ465397-DQ465404
http://doi.org/10.1016/J.ANTIVIRAL.2017.01.012	KT323979, KT323980, XM_003124230.2, NM_001201382, ACZ63406.1, NP_001159962.1
http://doi.org/10.1128/JVI.02125-16	CY076720-CY076727
http://doi.org/10.1111/IRV.12437	KJ175112 to KJ175143
http://doi.org/10.1016/J.VIRUSRES.2017.01.010	KP229773.1, EF413172.1, EF413173.1, EF413174.1, EF413175.1, EF413176.1, EF413178.1, AY463798.1 ,AY487249.1, AY487251.1, AY487250.1, KT037087.1, KT037088.1, KT037089.1, KT037090.1, KT037091.1, KC928078.1, KT037092.1)
http://doi.org/10.3201/EID2302.161374	CY207245–CY207252
http://doi.org/10.1128/JVI.01763-16	Table within article
http://doi.org/10.1155/2017/2978718	KM392227
http://doi.org/10.1016/J.VIRUSRES.2016.09.002	AFQ90538, AFQ90539, AFQ90540, AFQ90541, ADD84685
http://doi.org/10.1016/J.ANTIVIRAL.2016.05.006	FN434457-FN434464
http://doi.org/10.1016/J.FOODCHEM.2015.07.082	DQ297663, AF039170, NC_006853
http://doi.org/10.3389/FMICB.2016.01068	KX269879- KX269886
http://doi.org/10.1007/S11262-016-1303-4	KU177210-17
http://doi.org/10.1007/S12026-015-8772-8	EF635006
http://doi.org/10.1007/S12250-015-3709-X	AF506822
http://doi.org/10.1016/J.VIROL.2016.02.002	GQ166207,GQ166209, GQ166211, GQ166213, GQ166215, GQ166217, GQ166219, GQ166221

http://doi.org/10.1128/JVI.02718-15	NM_001128473.1, XM_003124230.2, NM_001246214.1, NM_001201382.1, NM_001244363.1, NM_001204395.1, NM_001001869.1, EU647216.1 ,NM_001005351, NM_001243452.1, NM_001161755, M65087.1, NM_213817.1, NM_001031790, GACC01000376.1, XM_003127919.2, XM_005665358.1, NM_001128473, NM_001128474
http://doi.org/10.1007/S00705-016-2819-6	Figure within article
http://doi.org/10.1016/J.MEEGID.2016.01.009	KC412634, KU360085
http://doi.org/10.1016/J.VETMIC.2016.01.005	EF413173, K00992-M57477
http://doi.org/10.1093/INFDIS/JIV399	Table within article
http://doi.org/10.1128/JVI.01457-16	Table within article
http://doi.org/10.1128/JVI.01551-15	FJ410137-133 FJ410144
http://doi.org/10.1128/JVI.02332-15	AF250356, KC209515
http://doi.org/10.1128/JVI.01188-16	FJ998208, FJ966082, GQ132146, FJ998214, FJ969517, GQ132164, FJ998211, FJ969513 ,GQ132174, FJ998217, FJ969512, GQ132169, FJ998220, FJ969514, GQ132182, FJ998223, FJ969515, GQ132151, FJ998226,
http://doi.org/10.1128/JVI.00119-16	ACR54994.1
http://doi.org/10.1007/S11262-015-1245-2	KT589138-321, KF260937, KF260695, KP413914, AB701294, KF357765, CY146750, KF259444 ,CY185437, KP413912, KP413913, LC028079, AB701294, JQ906577, KJ439878, JN605377, KM222576
http://doi.org/10.1038/SREP17010	EU708726, GQ475526, GQ330474, GQ461593
http://doi.org/10.1038/NATURE15379	CY184674–CY185309
http://doi.org/10.1371/JOURNAL.PONE.0138650	CY058516 to CY058523, GQ906800—GQ906802, GQ454863, GQ454864, GQ454865, GQ280121, GQ454866, GQ280122 ,GQ454867, GQ454868, EF467818- EF467824, EF467817, CY121687, CY121686, CY121685, CY121680, CY121683, CY121682, CY121681,
http://doi.org/10.1099/VIR.0.000184	LC053455, KM887144, AF353511.1
http://doi.org/10.1038/SREP12507	KR030166, HQ880611– HQ880618
http://doi.org/10.1371/JOURNAL.PONE.0133795	Table within article
http://doi.org/10.1016/J.VACCINE.2015.05.011	JN638733, JQ070800
http://doi.org/10.1038/SREP10651	PRU87392, EF6410081
http://doi.org/10.1016/J.VETMIC.2015.02.028	KM386876-KM386883
http://doi.org/10.1016/J.DCI.2014.10.008	XM_001928724, XM_001928671, CY083005, JN621781.1, JN621782.1, HQ200930.1
http://doi.org/10.1099/VIR.0.000058	AAD17229.1, ABP51969.1, ABI84534.1, BAF43460.1, CAD37074.1, AAA43099.1, AAA96134.1
http://doi.org/10.1371/JOURNAL.PONE.0122812	KJ577146-KJ577153, KJ577154-KJ577161, KJ577186-KJ577193, CY121792

http://doi.org/10.1038/NCOMMS7696	AHB20822-AHB20891, AHB20919-AHB23256, AHB23664-AHB23860, AHB24418-AHB24833, AHB82076-AHB82086
http://doi.org/10.1016/J.VETMIC.2014.12.021	FJ966082.1, GQ162170.1, GU480922.1, GU984405.1, GU984411.1
http://doi.org/10.1128/JVI.03355-14	JN409388-95, JN409420-27, JN409436-43, KP270886-93
http://doi.org/10.1186/S12985-015-0243-2	KJ013179-KJ013194
http://doi.org/10.1016/J.VETMIC.2014.11.031	HQ541664–HQ541671, HQ541640–HQ541647
http://doi.org/10.1016/J.TIM.2014.12.002	CCB78518, AHJ57400, AFV31481, ACY82404, ADW93781, AIO11674
http://doi.org/10.3201/EID2107.140981	KJ623706– KJ623713
http://doi.org/10.1016/J.ANTIVIRAL.2015.02.004	KP100813- 175KP101000, KP412321-KP412342
http://doi.org/10.1128/JVI.00459-15	Table within article
http://doi.org/10.1128/JVI.00840-15	KR699644-KR701609
http://doi.org/10.1128/JVI.00059-15	CY174077, CY174077
http://doi.org/10.1016/J.VIRUSRES.2015.09.003	KF683611–KF683618
http://doi.org/10.1016/J.VIRUSRES.2015.04.015	HQ315835, KM453698
http://doi.org/10.1007/S00705-015-2337-Y	HM998911-HM998918, HM998919-HM998926, KP081530-KP081537
http://doi.org/10.1186/S12985-014-0194-Z	KF840476-KF840478, KJ413857-KJ413933
http://doi.org/10.1186/S13567-014-0085-8	AF038150], JP011578], JP007133, JP006891, JP006358
http://doi.org/10.3390/V6051876	AB705392.1, JN786103.1, X01472.1, AJ009736.1, X03431.1, DQ887187.1, NM_017676.2, M32662.1 ,JN592050.1, HM143845.1, AF500296.1
http://doi.org/10.1128/MBIO.00031-14	KF425652-KF425658, KF425659-KF425665, KF425666-KF425672
http://doi.org/10.1371/JOURNAL.PONE.0089529	AY338458, CY076723
http://doi.org/10.1016/J.VIRUSRES.2013.10.022	GQ918133
http://doi.org/10.1016/J.VACCINE.2013.11.010	KF420205-KF420207, DQ176019, JN654459
http://doi.org/10.1128/JVI.03805-13	Figure within article
http://doi.org/10.1128/JVI.01080-14	Table within article
http://doi.org/10.1128/JVI.01327-14	KM027428-KM029955
http://doi.org/10.1099/VIR.0.067926-0	GQ280797

http://doi.org/10.1155/2014/598732	KF823970-KF823977, KF309210-KF309217, KF309218- KF309225, KF309226-KF309233, KF309234-KF309241, AY032626, EU807840, EF112445 ,JQ804986, HQ315836, KJ523894, KJ523895, KJ523896, KJ523897
http://doi.org/10.1128/JVI.03158-13	HQ533864, HQ533866,, HQ533868, HQ533871, HQ533872, HQ533874, HQ533877, HQ533879 ,JF951848-JF951855, JQ695860-JQ695883, KF918700-KF918707
http://doi.org/10.1016/J.MEEGID.2014.04.008	AY382481
http://doi.org/10.1007/S11262-014-1086-4	HQ315836, PRU87392, AF066183, AY424271, DQ988080
http://doi.org/10.1089/VIM.2014.0036	HQ650833, DQ499825
http://doi.org/10.1002/EJI.201344005	ABI47980, ABB88367, ABB20301, AAA43095, AF073181, H7N2NSB
http://doi.org/10.1016/J.TIM.2014.01.010	ABA55038.1, ABA55039.1, ABW36320.1, ABA55040.1, AGG82783.1, AAD17229.1, AAV48837.1, AAF77036.1 ,AAN06597.1, AAN06598.1, AAK14368.1, AAK14369.1
http://doi.org/10.1016/J.VETMIC.2013.09.007	Table within article
http://doi.org/10.1111/IRV.12193	Table within article
http://doi.org/10.1007/S00705-013-1771-Y	KC683516-KC683531
http://doi.org/10.1111/IRV.12105	KC142126–32<br>Table within article
http://doi.org/10.1016/J.VIROL.2013.08.004	CY130717.1, 11SW347, CY131957.1
http://doi.org/10.1186/1743-422X-10-290	Table within article
http://doi.org/10.1016/J.VIRUSRES.2013.05.017	EF413173
http://doi.org/10.1007/S00705-013-1672-0	S77541, EF413172, EF413173, EF413174, EF413176, EF413178
http://doi.org/10.1016/J.MEEGID.2013.04.034	KC209503–KC209519
http://doi.org/10.1016/J.VETIMM.2013.04.004	HE774666, HE774667, HE774668,, HE774669, HE774670, HE774671, HE774672, HE774673 ,GQ464405–GQ464411, GQ168897
http://doi.org/10.1016/J.JCV.2013.03.011	A00857129, 00857318a, 00857318a, A01049091, A01134524, A00857318a
http://doi.org/10.1128/JVI.00804-13	CY130717, CY130719, CY131957, CY131959,, CY131053, CY131055, CY130909,, CY130911, ,KF007981-KF008000
http://doi.org/10.1128/JVI.00979-13	. KC845536, KC845537, AAA38674), AAB59662, AAB53776, P01862, KC845538, KC845539 ,KC845534, KC845535
http://doi.org/10.1128/JVI.00373-13	Table within article
http://doi.org/10.1016/J.MEEGID.2013.03.006	Figure within article
http://doi.org/10.1186/1297-9716-44-41	JF275917-JF275924, JF275933-JF275940, JF275941-JF275948, JF275925-JF275932, KC683492-KC683499
http://doi.org/10.3892/IJMM.2013.1274	HM014332.1
http://doi.org/10.1016/J.VETMIC.2012.10.006	JF714149–JF714156<br>Figure within article
http://doi.org/10.1002/JMV.23482	JQ693686–JQ693765

http://doi.org/10.1128/JVI.02507-12	HQ111361 to HQ111368
http://doi.org/10.1016/J.MEEGID.2012.09.021	HQ541648– HQ541655, HQ541680–HQ541711
http://doi.org/10.1177/1040638712466554	FS638306, EU139827, FJ638306
http://doi.org/10.1126/SCIENCE.1239844	KF021594 to KF021601
http://doi.org/10.1128/JVI.02386-12	AY032626, EF641008.1
http://doi.org/10.1016/J.VIROL.2013.08.010	DQ497729
